# Treatment of menopausal symptoms by an extract from the roots of rhapontic rhubarb: the role of estrogen receptors

**DOI:** 10.1186/1749-8546-5-7

**Published:** 2010-02-19

**Authors:** Günter Vollmer, Anja Papke, Oliver Zierau

**Affiliations:** 1 Molekulare Zellphysiologie & Endokrinologie, Fachrichtung Biologie, Technische Universität Dresden, Zellescher Weg 20b, 01217 Dresden, Germany

## Abstract

A dry extract from the roots of rhapontic rhubarb (extract *Rheum rhaponticum *(L.); ERr) has been commercially available in Germany for over two decades to treat menopausal symptoms. However, the molecular basis of its clinical effectiveness remains obscure. This article reviews the *in vitro *and *in vivo *data of its estrogenic actions, particularly those mediated by estrogen receptor-β (ERβ).

## Background

**Figure 1:**
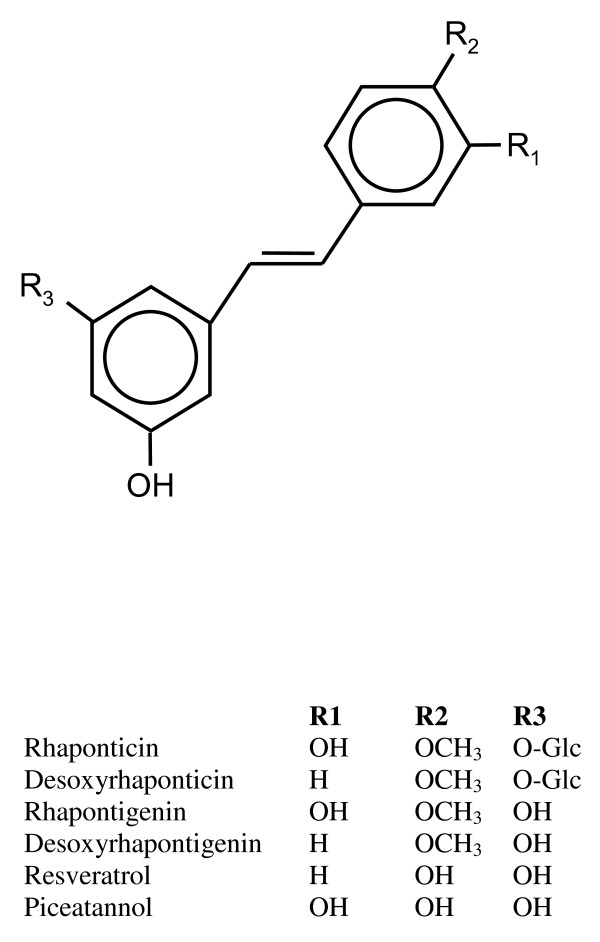
**Structure of hydroxystilbenes**.

A dry extract from the roots of rhapontic rhubarb (extract *Rheum rhaponticum *(L.); ERr; *Dahuang*) consists mainly of rhaponticin (<90%) and aglycones (5%) of rhaponticin and desoxyrhaponticin (Figure 1). In plants, these natural hydroxystilbene compounds share a common biosynthetic pathway with resveratrol which is the first stilbene compound produced from p-coumaroyl-CoA catalysed by a selective stilbene synthesis. All other hydroxystilbenes in *Rheum rhaponticum *are derivatives of resveratrol [1].

No adverse events have been observed in human applications of ERr although the synthetic estrogen diethylstilbestrol with a structural similarity of hydroxystilbenes exerts deleterious transplacental effects in humans [2,3]. In both sexes of the dogs, a dosage of 1000 mg/kg body weight per day produced no adverse events [4].

## Effectiveness of ERr in treating menopause

Since 1993 when ERr was first used in human to treat menopausal symptoms, and before when it used to treat women with child bearing potential suffering from oligomenorrhea or amenorrhea, no severe adverse events have been reported [5]. Ovarian failure during menopausal transition evokes a variety of symptoms. Postmenopause is established after 12 months of amenorrhea [6]. Perimenopause is the period of time (up to five years) prior to the last menstruation. About 80% of menopausal women suffer from hot flushes, sweating, heart complaints, sleep disturbances, depression, anxiety, physical/mental exhaustion, sexual problems, urinary tract complaints and vaginal dryness.

While hormone therapy (HT) effectively relieves menopausal symptoms, many women seek alternative treatments due to increased risks for breast [7] and endometrial cancer [8] associated with HT, as well as venous thromboembolism. The effectiveness of ERr to treat menopausal symptoms was validated by a 3-month prospective randomized controlled trial on 54 perimenopausal women with climacteric complaints receiving a daily dose of 4 mg of ERr. The treatment with ERr after 12 weeks significantly reduced the Menopause Rating Scale II symptoms as compared to control. After four weeks of treatment, the number and severity of hot flushes were decreased. After 12 weeks the overall menopause-related quality of life was significantly better in ERr-treated women than in those who received placebo. Importantly, no difference was observed in gynecological findings including endometrial biopsies, bleeding weight, blood pressure and tested laboratory safety parameters. No adverse events were detected in relation to ERr intake [9]. ERr is effective in reducing anxiety in perimenopausal women according to the Hamilton Anxiety Scale [9].

Continued intake of ERr for 48 and 96 weeks demonstrated long-term safety of ERr as no endometrial hyperplasia was detected, and no adverse events related to ERr occurred [5].

ERr reduces the occurrence and severity of climacteric symptoms during perimenopause in women and improves their health-related quality of life [9,10].

## Estrogenic activities of ERr

Clinical observations suggest that the mode of action of ERr is similar to that of hormones (i.e., estrogens). Estrogenic activities of ERr were assessed at biochemical, molecular and cellular as well as organism levels. Ligand binding assays and uterotrophic assays are two major methods to investigate the estrogenic activities of ERr. Bound estrogen receptors (ERs) usually modify transcriptional activity of target genes in a target cell. This process may be investigated by reporter gene assays in which a target cell is transfected by an expression plasmid for the ER of interest and a reporter gene construct, e.g. luciferase, whereby functional activity of ERs is quantified in target cells [11]. Finally, an organism as a whole has a high metabolic capacity. A compound given to an organism may be subject to its metabolism thereby rendering its activity. Therefore, results obtained *in vitro *must be verified *in vivo*. Ovariectomized female rodent is the recommended animal model for estrogen-related studies [12,13]. If a compound is administered to ovariectomized rodents and exhibits estrogenic activities, uterine growth will be stimulated [14,13].

### Ligand binding assays of ERr

Ligand binding affinity for rhapontigenin and desoxyrhapontigenin, the aglycones of the major constituents of ERr, was determined by fluorescence polarisation according to Mueller *et al*. [15]. As the assay does not tolerate complex substance mixture of the entire extract, the ligand binding affinity of the total extract cannot be determined. Both substances exhibit a weak binding affinity to either estrogen receptor α (ERα) or estrogen receptor β (ERβ) with a slight preference for ERβ (Table 1) which is typical in a number of phytestrogens [11].

**Table 1:** Relative binding affinities of major constituents of ERr and estradiol

Substance	IC_50 _ERα	IC_50 _ERβ	Fold preference for ERβ
Estradiol	8 × 10^-9^	6.7 × 10^-9^	0.84
Trans-rhapontigenin	1.2 × 10^-5^	5.6 × 10^-6^	2.14
Desoxyrhapontigenin	2.6 × 10^-5^	2.8 × 10^-5^	1.1

Note:

Relative binding affinities of trans-rhapontigenin and desoxyrhapontigenin were assessed by a fluorescence polarization assay. IC_50 _values of compounds in comparison to estradiol and relative preference for the receptor subtypes are shown.

This weak binding preference suggests that the stimulation of cell-based reporter genes is equally potent for both receptors. Rhapontigenin and desoxyrhapontigenin almost only act on ERβ. ERr extract activates reporter gene activity through ERβ in HEC-1B endometrial adenocarcinoma cells and an endometrial specific reporter gene construct (mC3-tk-Luc) [16]. In a study using bone-derived U2OS cells and a reporter gene construct ((ERE)_2_-t-Luc), a weak activation of the reporter gene through ERα by the ERr extract and the two hydroxystilbenes was detected, whereas a strong activation of ERβ-dependent reporter gene by the ERr extract and the two hydroxystilbenes was observed [17].

The strong ERβ specificity observed in cell-based assays cannot be explained by the weak binding preference. A reasonable explanation might be due to recruitment of co-factors upon ligand binding [18]. Moreover, two other factors may be attributed to the differences in ligand binding and transcriptional activation. Firstly, there is a second binding site for substances such as tamoxifen in the co-activator groove of the ERβ, and thus direct antagonistic co-activator receptor interaction is possible [19,20]. Whether this effect can be mimicked by ERr or its constituents remains to be investigated. Secondly, ligand binding does not affect the mobility of ERβ, but significantly influences the mobility of ERα, in the studies using pure anti-estrogen fulvestrant or the selective estrogen receptor modulator (SERM) raloxifen [21], whether and how ERr and/or its constituents influence(s) nuclear mobility upon binding is still not clear.

## Rodent uterotrophic assays of ERr

The absence of an increased uterine wet weight in the in vivo uterotrophic assay for estrogenicity [14,13] in ovariectomized rats indicates the safety of herbal extracts in terms of proliferative stimulation within the uterus. However, the assay data should not be directly extrapolated to human because ovariectomized rodents after a hormonal decline of 10-14 days are almost void of estrogens, while women in menopausal transition and postmenopause, despite the decline of ovarian estrogen production, have measurable estrogen levels in their circulation due to extraovarian estrogen production.

We performed two versions of the 3-day uterotrophic assay. In version 1, we tested four doses of ERr in comparison to the positive control estradiol with 0.1 mg/kg body weight per day as a therapeutically dose and three pharmacological doses (1, 10 and 100 mg/kg body weight per day) for safety considerations. In version 2, the same dose range of ERr was tested in a combinatorial treatment in the presence of low doses of estrogens (0.5 μg/kg body weight per day) to mimic the hormone levels in menopausal women [22]. Our results demonstrated two key features of ERr in the uterus of ovariectomized rats: (1) none of the tested doses of the extract if given alone stimulated uterotrophy (Table 2) or marker gene expression associated with proliferative events (data not shown); (2) treatment of ovariectomized rats with combinations of low dose estradiol and ERr dose dependently counteracted estradiol induced uterotrophy, i.e., the treatment resulted in a decrease in uterus wet weight (Table 2). This is interesting because both the uterotrophic response itself and the proliferation of the rat uterus are mediated by ERα primarily through a tethering mechanism with activator protein 1(AP-1) transcription factors [23].

**Table 2:** Relative uterotrophic and anti-uterotrophic activities

ERr 731 (μg/kg BW/d)	Estradiol (mg/kg BW/d)		
	0	0.5	4
0	100	211.5 ± 42.7***	576.6 ± 220***/623.8 ± 35.6***
0.1	75.5 ± 9.2/97.3 ± 24.9	189.3 ± 46.1***	-/-
1	85 ± 8.7	162.8 ± 35.1***	-/-
10	103.3 ± 15.2	182.5 ± 31.7***	-/-
100	122.9 ± 13.7/104.2 ± 19.6	130 ± 18.1*/^++^	-/-

Note:

The mean ± SD of uterine wet weights expressed as percentage of untreated controls are shown. If two values are shown, they represent means ± SD from two independent experiments.

*P < 0.05, ***P < 0.001: statistical significance (Student's t-test) if compared to untreated controls

^++^*P *< 0.01: statistical significance (Student's t-test) if compared to low dose estradiol treatment (0.0005 mg/kg BW/d)

Both *in vitro *and *in vivo *data support that ERr and/or its constituents exhibit(s) SERM-like properties. This hypothesis is supported by the results of a recent study in which three synthetic SERMs were comparatively evaluated. Overall, they exhibited mixed agonistic/antagonistic properties but shared one common feature, namely the reduction of the estradiol-stimulated uterotrophic response [24]. Another study on synthetic SERM discovered groups of compounds which functionally resemble the ERr by exhibiting a binding preference for ERβ and an anti-uterotrophic response in the uterotrophic assay [25]. Thus, it seems justified to associate ERr with functional SERM-like properties.

## Plants containing hydroxystilbenes in Chinese medicine

Plants, plant parts or seeds containing hydroxystilbenes are used in Chinese medicine. *Semen Astragali *(SA) is a traditional Chinese tonic which may improve conditions such as hypertension and liver fibrosis [26-28]. Some Chinese herbs such as *Polygonum cuspidatum*, *Rheum tanguticum*, *Rheum officinale *and *Rheum coreanum *have been used to alleviate menstrual and postmenopausal symptoms. *Polygonum cuspidatum *exhibited estrogenic activities from the anthrachinone emodin and the modified resveratrol hydroxystilbenes [29]. These studies suggest a positive role of hydroxystilbenes in the management of menopausal symptoms.

## Conclusion

Neither clinical nor experimental studies observed adverse events of ERr, e.g., uterine and endometrial growth and proliferation. Moreover, ERr exerts SERM-like activities. For function ERβ seems to be the more important ER which in turn has been proven to mediate e.g., anti-anxiety effects [30].

## Competing interests

The authors declare that they have no competing interests.

## Authors' contributions

GV reviewed the clinical data and all in vitro data. AP and OZ contributed to the review of the available animal data. All authors read and approved the final version of the manuscript.
